# Striatal Neurodevelopment Is Dysregulated in Purine Metabolism Deficiency and Impacts DARPP-32, BDNF/TrkB Expression and Signaling: New Insights on the Molecular and Cellular Basis of Lesch-Nyhan Syndrome

**DOI:** 10.1371/journal.pone.0096575

**Published:** 2014-05-07

**Authors:** Ghiabe-Henri Guibinga, Nikki Barron, William Pandori

**Affiliations:** Department of Pediatrics, Division of Genetics/Rady Children’s Hospital, University of California San Diego School of Medicine, La Jolla, California, United States of America; University of Louisville, United States of America

## Abstract

Lesch-Nyhan Syndrome (LNS) is a neurodevelopmental disorder caused by mutations in the gene encoding the purine metabolic enzyme hypoxanthine-guanine phosphoribosyltransferase (HPRT). This syndrome is characterized by an array of severe neurological impairments that in part originate from striatal dysfunctions. However, the molecular and cellular mechanisms underlying these dysfunctions remain largely unidentified. In this report, we demonstrate that HPRT-deficiency causes dysregulated expression of key genes essential for striatal patterning, most notably the striatally-enriched transcription factor B-cell leukemia 11b (Bcl11b). The data also reveal that the down-regulated expression of Bcl11b in HPRT-deficient immortalized mouse striatal (STH*dh*) neural stem cells is accompanied by aberrant expression of some of its transcriptional partners and other striatally-enriched genes, including the gene encoding dopamine- and cAMP-regulated phosphoprotein 32, (DARPP-32). Furthermore, we demonstrate that components of the BDNF/TrkB signaling, a known activator of DARPP-32 striatal expression and effector of Bcl11b transcriptional activation are markedly increased in HPRT-deficient cells and in the striatum of HPRT knockout mouse. Consequently, the HPRT-deficient cells display superior protection against reactive oxygen species (ROS)-mediated cell death upon exposure to hydrogen peroxide. These findings suggest that the purine metabolic defect caused by HPRT-deficiency, while it may provide neuroprotection to striatal neurons, affects key genes and signaling pathways that may underlie the neuropathogenesis of LNS.

## Introduction

A number of important and generally inadequately-treated human neurological diseases results from abnormalities in the function of the striatum, a region of the forebrain which plays an important role in the coordination of movement, cognition and behavior. Striatal function is severely perturbed in prominent disorders like Huntington disease (HD), as well as in less common ones such as Lesch-Nyhan syndrome (LNS), a neurodevelopmental disorder caused by mutations in the gene encoding the purine reutilization enzyme hypoxanthine guanine phosphoribosyl transferase (HPRT). This syndrome is characterized by an array of neurological dysfunctions that include mental retardation, dystonia, choreoathetosis, spasticity, and compulsive uncontrollable self-mutilation [1], [2]. On the contrary to HD whose one of main neuropathogenic characteristic is the degeneration of striatal neurons, the neural defects in LNS occur without noticeable striatal cell death. Nonetheless, LNS patients show marked loss of striatal dopaminergic fibers [3], [4]; also, the surrogate model for LNS, the HPRT knockout (HPRTKO) mouse displays marked losses in striatal dopamine content and abnormal striatal architecture [5]–[8].

While the HPRT enzyme is mostly known for its purine metabolic housekeeping functions and is expressed ubiquitously in all the cells, its connection to neuronal function is unclear. We and others have recently presented evidences that HPRT-deficiency leads to perturbations in the expression of transcription factors essential for neurogenesis [9], [10], among them, the basic helix-loop-helix (bHLH) transcription factor Ascl1 (also known as Mash1). Ascl1 belongs to the *achaete-scute* family and is important for the successful differentiation of several types of neurons. In the context of dopaminergic differentiation, we have previously reported that the expression of Ascl1 and two of its neural transcriptional partners, Neurogenin 2 (Ngn2) and Nurr1 is markedly down-regulated in HPRT-deficient neuron-like cells [9], [10]. Interestingly, Ascl1 is also known to be crucial for the differentiation and specification of GABAergic neurons that make up the forebrain, where in concert with other transcription factors it orchestrates proper striatal patterning in vivo [11], [12].

In the present study we hypothesized that given the known involvement of Ascl1 in HPRT-deficiency and its role in guiding striatal patterning, its dysregulation may underlie striatal function deficits in LNS/HPRT-deficiency. Therefore in an attempt to define some of the molecular and cellular basis of striatal defects in HPRT-deficiency, we have examined the expression of several striatally-enriched genes in HPRT-deficient mouse striatal STH*dh* cells, principally the transcription factor B-cell leukemia 11b (Bcl11b) (also known as Citp2). Bcl11b plays a crucial role in striatal neurodevelopment and is highly enriched in medium spiny neurons (MSNs) that constitute about 90% of neuronal cell population in the striatum [13]. We demonstrate that Bcl11b expression is markedly down-regulated in HPRT-deficient striatal STH*dh* cells and in the striatum of adult HPRTKO mouse. We also show dysregulated expression of the protein phosphatase 1 regulatory (inhibitor) subunit b (*Ppp1r1b*) gene, encoding the striatally-enriched dopamine- and cAMP-regulated phosphoprotein 32, (DARPP-32), along with the altered expression of Thr34-phosphorylated DARPP-32 in HPRT-deficient striatal cells and tissues. Finally, we present evidence that HPRT-deficiency up-regulates the expression of the brain-derived neurotrophic factor (BDNF)/TrkB pathway, and in so doing confers superior protection to HPRT-deficient striatal cells against reactive oxygen species (ROS)-mediated cell death induced with hydrogen peroxide (H_2_O_2_). Collectively, the findings of this study identify new cellular and molecular effectors for HPRT-deficiency-mediated striatal dysfunctions and propose that the targeting DARPP-32/BDNF/TrkB may be a therapeutic strategy for LNS.

## Materials and Methods

### Cells

Immortalized wild-type ST*Hdh^Q7^* striatal cells have been previously described [14] and were kindly provided by Dr. Albert La Spada (UCSD). The striatal cells were grown at 33°C in Dulbecco’s modified Eagle’s medium (DMEM) supplemented with 10% fetal bovine serum (FBS), 1% non-essential amino acids, 2 mML-glutamine in 5% CO_2_ atmosphere. We also selected human control and LNS fibroblasts exhibiting HPRT enzymatic activity consistent with severe LNS phenotype. These fibroblasts obtained from a tissue bank have been previously described [10], [15], and were kindly provided by Dr. Jinnah, (Emory University, Atlanta, GA). The primary human fibroblasts were cultured in DMEM medium supplemented with 10% FBS and 50 µg/ml penicillin/streptomycin.

### Animals

C57BL/6J and C57BL/6J-*Hprtbm3 (HPRTKO)* mice have been described before [7], [16], [17] and were obtained from Jackson Laboratories (Bar Harbor, ME). Wild type and HPRTKO mice are maintained in individual cages at the University of California San Diego’s animal care facilities, where they are kept on a 12-h light–dark cycle with free access to food and water. Brain tissues encompassing the striatum of at least three adult male wild-type (WT) or three adult male HPRTKO mice were collected and sonicated in homogenizing buffer (1% SDS, 50 mM NaF). The protein concentration of the homogenate was determined by BCA protein assay and processed for immuno-blot as described below. Alternatively, the striatal tissues were rapidly frozen under powdered dry ice for RNA extraction using RNA extraction procedures indicated below. All animal procedures were conducted in accordance of the published guidelines from UCSD (institutional) and the National institute of health (Federal). In addition all the animal experimentation complied with the ARRIVE (Animal research of reporting in vivo experiments) guidelines. All the experiments were approved by UCSD Institutional Animal Care and Use Committee (IACUC).

### HPRT and Luciferase Small Hairpin Oligonucleotides and Knockdown Vectors

The short hairpin RNA (shRNA) sequences against the luciferase and *Hprt* genes were selected and prepared as previously described [9].

### HPRT Knockdown and Selection of HPRT-deficient Striatal Cells

Cells were infected at a multiplicity of infection (MOI) of 1 with the knockdown retroviral vectors, retrosh2hprt or control vector retroshlux. Infected cells were grown for 10 days in complete DMEM medium containing 3 µg/ml of puromycin. Bulk cultures were re-plated and maintained in DMEM without puromycin selection for additional 7 days, after which cells were examined for HPRT expression by QPCR, western-blot and by transfer to the culture medium containing 250 µM 6-thioguanine (6-TG) (SIGMA). The cells infected with the HPRT knockdown vector retrosh2hprt showed unimpeded growth and expansion in 6-TG while the control cells infected with retroshlux were unable to grow in 6-TG (data not shown).

### Total RNA Isolation and Quantitative PCR Analysis

Total RNA was isolated using PureLink™ RNA Mini kit for control and HPRT-deficient cells, according to the manufacturer’s instructions (Ambion/Life Technologies). RNA quantity, quality and integrity were evaluated using the Nanodrop (Thermo scientific). 500 ng of RNA were submitted to reverse transcription reaction using Qiagen miScript reverse transcription kit according to manufacturer’s instructions (Qiagen). The synthesized cDNA was then run by qPCR analysis using a Qiagen miScript kit. All PCR primers used for this study are listed in Table S1. The Quantitative PCR was carried out twice in duplicate or triplicate,using the Opticon 2 system DNA engine (BioRad) in a total reaction volume of 20 µl and in the presence of 200 nM of each of the primers. Primers specific for the glyceraldehyde-3-phosphate dehydrogenase (GAPDH) were used as normalization controls. In order to verify the specificity of each amplicon, a melting curve analysis was included at the end of each run. Differences of expression between control and knockdown groups were calculated by normalizing Ct values of the test genes to the Ct values of an endogenous control (GAPDH). The fold change or difference in mRNA expression was calculated using the equation 2-ΔΔCt [18]. The statistical significance of variation of expression was assessed by comparing ΔCt of each group by student, t-test.

### Dopamine Receptor 1 (D1R) agonist SKF38393 and BDNF Treatment

Control and HPRT-deficient striatal cells were seeded in 6-well plate (2×10^5^ cells per well) and in serum-free conditions. The cells were subsequently treated with vehicle PBS (control) and up to 50 µM SKF38393 (From Sigma) for 15 min in serum free conditions. The reaction was terminated by removal of the medium and the subsequent addition of mammalian protein extraction reagent (M-PER from Thermo-Scientific) and protease inhibitors cocktail (From SIGMA). Separately, control and HPRT-deficient striatal cells were also treated with PBS or BDNF (10 ng/ml) for 15 min. Cell lysate was recovered for both SKF38393 and BDNF treatments and processed for protein quantification and immuno-blot analyses.

### Hydrogen Peroxide Treatment

Control and HPRT-deficient cells were seeded in the conditions indicated above and treated with vehicle PBS control and 100 µM of hydrogen peroxide for at least 15 min; afterward the medium was removed and the cells were exposed to mammalian protein extraction reagent containing protease inhibitors. The cell lysate was recovered and processed for protein quantification and immuno-blot analysis.

### Immuno-blot

Cells were cultured and treated as indicated above. The cell lysates were centrifuged 15,000 g at 4°C for 10 minutes. Protein concentration assays of supernatant were carried out by BCA assay (Thermo Scientific). Subsequently, thirty micrograms of protein lysate was separated by reducing-Tricine-SDS-PAGE and the separated protein bands were transferred onto polyvinylidene fluoride (PVDF) membrane (Invitrogen) using Mini Trans-Blot Electrophoretic Transfer Cell (BioRad). Blotted PVDF membrane were blocked by blocking solution containing 5% bovine serum albumin in TBST (20 mM of Tris, 137 mM of NaCl and 0.1% of Tween-20 (SIGMA)) for one hour at room temperature. Immuno-detection of primary antibodies was carried out overnight at 4°C and signal amplification using horseradish peroxidase (HRP)-conjugated secondary antibody was performed at room temperature for one hour. The chemiluminescence reagent SuperSignal West Pico (Thermo Scientific) was used as signal amplification reagent. The x-ray films were developed by SRX101A film processor (Konica Minolta, Motosu-shi, Gifu-ken). All primary and secondary antibodies were diluted in the aforementioned blocking solution. The primary polyclonal rabbit antibody against GAPDH (2118L), DARPP-32 (#2302S) and p-DARPP-32 (Thr34)(#12438S) were obtained from Cell Signaling Technology (cellsignaling.com). Bcl11b (ab28448) and Ascl I (sc-13219) antibodies were obtained from AbCAM and Santa Cruz biotechnology, respectively. Finally, antibodies against BDNF (AB1534SP), TrkB (#4638S) and phospho-TrkB (#4638S) were purchased from EMD-Millipore and Cell Signaling Technology (cellsignaling.com). All antibodies were used at dilutions ranging from 1∶500 to 1∶1000. The secondary IgG antibodies against the corresponding primary antibodies were labeled with horseradish peroxidase (HRP, from Cell signaling Technology, #7074S) and used at the dilution 1∶1000 to 1∶5000). The immuno-blot signal was quantified using densitometry Image J software according to the protocol published at http://openwetware.org/wiki/Bitan:densitometry. Protein expression of GAPDH was used as loading normalization control.

### SYTOX Fluorescent Staining

Control and HPRT-deficient cells were seeded in the conditions indicated above and treated with vehicle PBS control and 100 µM of hydrogen for at least four hour, afterward the cells were washed twice with 1X PBS and exposed to 100 nM of SYTOX green (Life Technologies) in 1X PBS for 10 min. Subsequently, the SYTOX dye was removed and the cells washed twice with 1X PBS and fixed with 4% paraformaldhyde for 3 min and then washed with 1X PBS. The resulting cells were mounted in Vectashield media containing 4′,6-diamidino-2-phenylindole (DAPI) (Vector Laboratories). Fluorescence was visualized using Olympus BX51 fluorescent microscope with BP72 Olympus acquisition camera.

### Plasmid Transfection

Cells were plated at 2×10^5^ cells per well in six well tissue culture plates. The following day the cells were transfected with an expression plasmid (pCMV6) containing the cDNA of mouse Bcl11b (variant 2, BC019503 from oriGENE) using “TRANSFECTENE” transfection reagent (Qiagen) according to the manufacturer’s instructions. A GFP expression vector was used as control. 48–72 hours after transfection, mRNA and proteins were extracted from the transfected cells and submitted to QPCR and immune-blot analyses. The transfected cells were also exposed to hydrogen peroxide and evaluated for TrkB signaling as well as Sytox-green mediated assessment of cell death.

### Data and Statistical Analysis

The experiments were carried out at least twice (on separate samples, i.e technical replicates), and where indicated, in duplicate or triplicate (experimental replicates). The data are reported as mean plus or minus (±) standard error (SEM). Statistical and data analyses were performed using Kaleidagraph graphing & data analysis software package (Synergy Software, Reading, PA). Student paired t-test was performed on experimental data involving a single variable (HPRT status, control vs HPRT-deficiency). Conversely, for data with more than single variables such as (HPRT, Bcl11b reconstitution status), a one way ANOVA with Tukey post-hoc test was also performed. For all analyses, statistical significance was set at p<0.05.

## Results

### Preparation of Control and HPRT-knockdown Striatal (STHdh) Cells and Down-regulated Expression of Ascl1 and other foRebrain Specific Transcription Factors in HPRT-Deficiency

**Figure 1:**
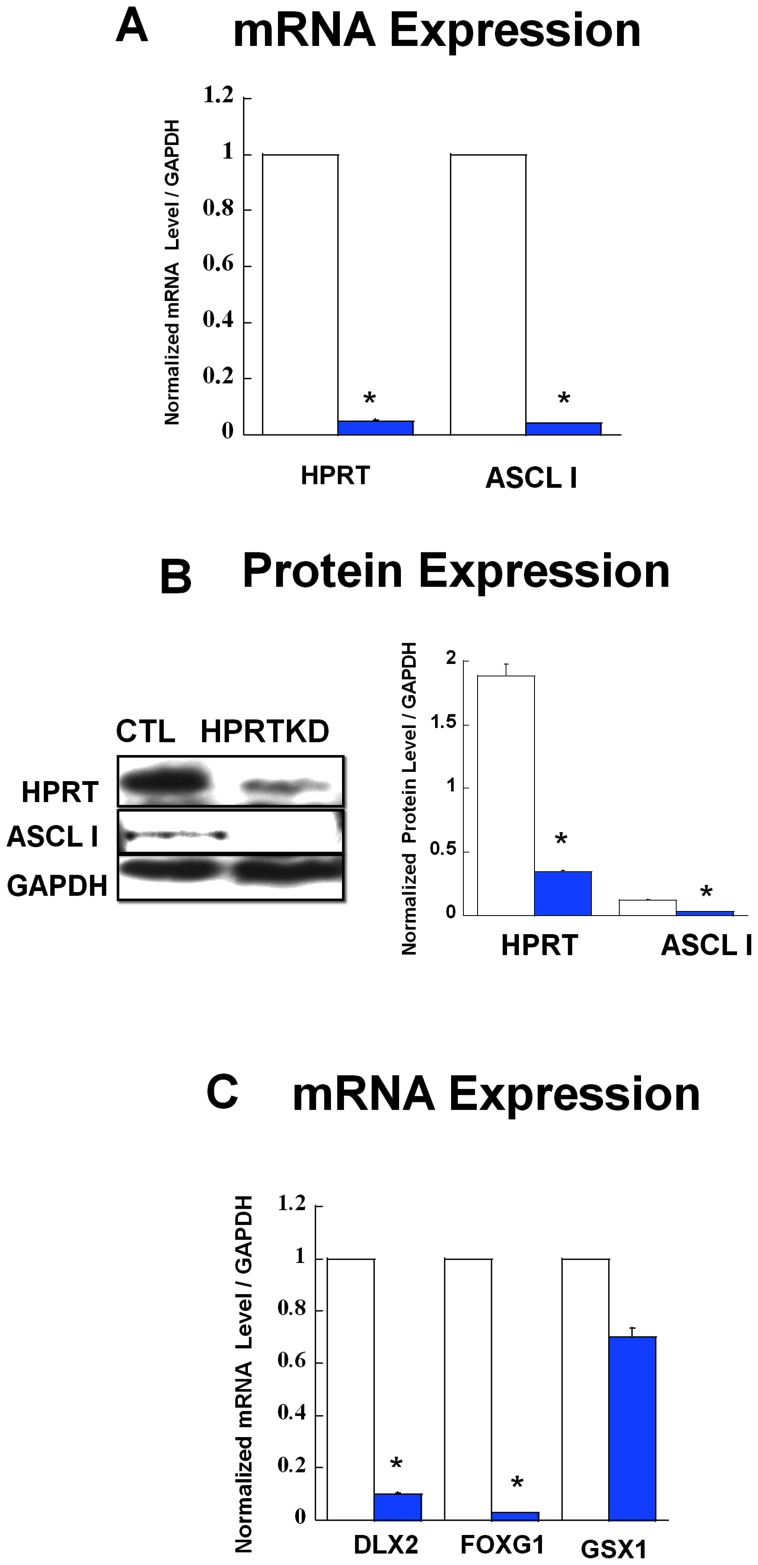
Gene & Protein expression for HPRT and ASCL1. (**A**) Gene expression for HPRT and ASCL1 in control (CTL, open bars) and HPRT knockdown (HPRTKD, closed bars) striatal STHdh cells. Bars represent mean ±SEM of duplicate PCR measurements carried out independently twice (n = 4). mRNA expression is normalized to GAPDH level (*P<0.05, t-test). (**B**) Immuno-blot and quantification of HPRT and ASCL1 protein expression in striatal STHdh cells. The quantification bar graphs are shown as means ± SEM (n = 3, *P<0.05, t-test). (**C**) Gene expression for DLX2, FOXG1 and GSX1 in control (CTL, open bars) and HPRT knockdown (HPRTKD, closed bars) Striatal cells. Bars represent mean ±SEM (n = 4) *P<0.05, t-test.

For this study we used immortalized mouse striatal neural (STH*dh*) stem cells, that are derived from striata of embryonic 14 day tissue and are well recognized cellular model for striatal studies [19], [20]. The ST*Hdh* cells were infected with lentiviral vectors encoding a small hairpin directed at the *Hprt* or luciferase (control) genes under the conditions previously described [21], [22]. Data show the efficiency of the *Hprt* gene knock down as illustrated in Figure 1A & 1B through quantitative PCR and immuno-blot analyses. The data reveal 96% HPRT gene and protein reduction (Fig. 1A & 1B); additionally, in order to confirm the HPRT-deficiency phenotype, the ST*Hdh* control and HPRT-knock down cells were expended for 72 hours in the cell culture medium containing 6-thioguanine [6-TG] (250 µM) under the conditions previously described [23]. Control cells show limited growth and noticeable cytotoxicity in presence of 6-TG, while HPRT knock-down cells were able to expand under the same conditions, indicating that they had become HPRT-deficient (data not presented).

We have previously demonstrated that HPRT-deficiency alters the expression of the neural transcription factor Ascl1 (Mash1) in human neuron-like cell lines [9], [10]. In the context of this present study, we wanted to confirm whether the transcriptional alterations of Ascl1 apply to the HPRT-deficient ST*Hdh* striatal cells. Our data show a marked and a significant reduction of *Ascl1* mRNA and protein level (Figure 1A & 1B). As further evidence that HPRT-deficiency may dysregulate striatal-neural specific programming, we also examined the expression level of three important transcription factors known to act alongside Ascl1 and drive striatal specification in the forebrain, DLX2, FOXG1 and GSX1 [12]. Our data show marked down-regulation of *Dlx2* and *Foxg1* gene expression in HPRT-deficient striatal cells relative to control, while the level of *Gsx1* gene expression is not statistically different between control and HPRT-deficient cells (Figure 1C).

### Down-regulated Expression of Striatal-specific Transcription Factor Bcl11b and other Striatally-enriched Genes in HPRT-deficient Striatal (STHdh) Cells and Tissues

**Figure 2:**
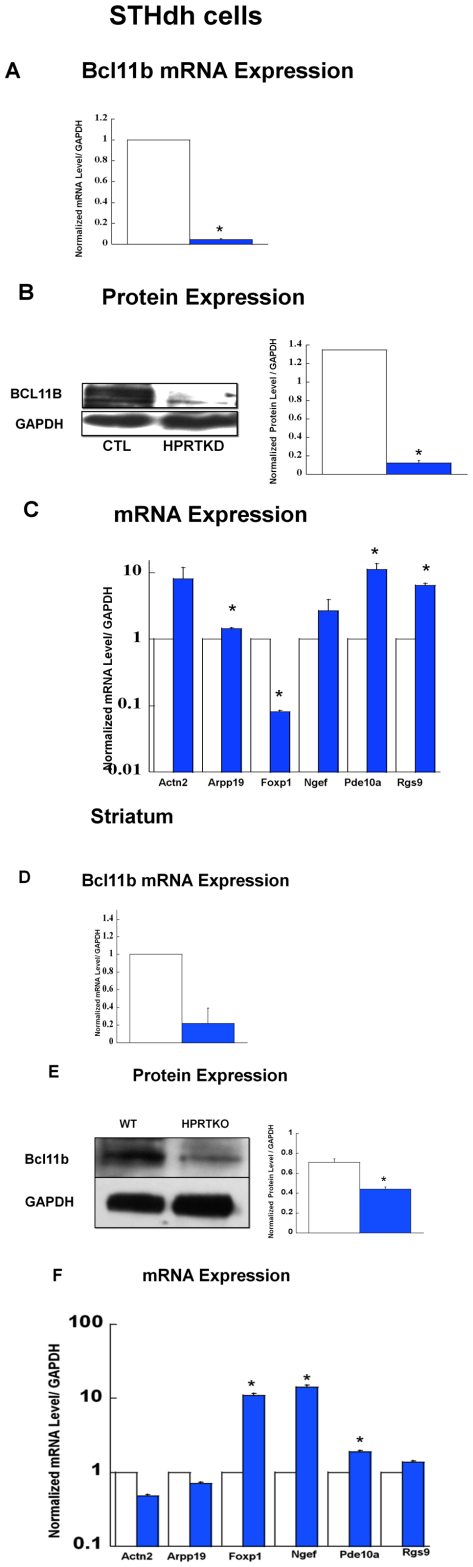
Gene and Protein Expression for Bcll11b. (**A**) Gene expression for Bcl11b in control striatal STHdh cells (CTL, open bars) and HPRT-knockdown (HPRTKD, closed bars). Bars represent mean ±SEM (n = 4). mRNA expression is normalized to GAPDH RNA level (*P<0.05, t-test). (**B**) Immuno-blot and quantification of Bcl11b protein expression in striatal cells. The quantification bar graphs are shown as means ± SEM (n = 3, *P<0.05, t-test). (**C**) Gene expression of Actn2, Arpp19, Foxp1, Ngef, Pde10a, and Rgs9 in control (CTL, open bars) and HPRT knockdown (HPRTKD, closed bars) striatal STHdh cells. Bars represent mean ±SEM (n = 4). *P<0.05, t-test. (**D**) Gene expression for Bcl11b in the striatum of wild-type (WT, open bars) and HPRT knockout (HPRTKO, closed bars) mice. Bars represent mean ±SEM (n = 4). mRNA expression is normalized to GAPDH RNA level (*P<0.05, t-test). (**E**) Immuno-blot and quantification of Bcl11b protein expression in the striatum of WT and HPRTKO mice. The quantification bar graphs are shown as means ± SEM (n = 3, *P<0.05, t-test). (**F**) Gene expression for Actn2, Arpp19, Foxp1, Ngef, Pde10a, and Rgs9 in control (Wild-type, open bars) and HPRT knockout mice (HPRTKO, closed bars) striatum. Bars represent mean ±SEM (n = 4). *P<0.05, t-test.

In order to assess further the striatal transcriptional regulation in HPRT-deficiency, we turned our attention on Bcl11b, a transcription factor which plays a central role in the development of striatal neurons, specifically MSNs that constitute the majority of striatal neuronal cells population [24]. Our data show a significant reduction of *Bcl11b* mRNA and protein level in HPRT-deficient STH*dh* cells relative to control (Figure 2A & 2B). The *Bcl11b* gene is enriched in the striatum and activates the transcription of several other striatally-enriched genes [25]. Therefore, we elected to analyze comparatively, the expression of several striatally-enriched genes believed to be regulated by Bcl11b (Foxp1, Rgs9, Ngef, Arpp19, Actn2 and Pde10A) in control and HPRT-deficient STH*dh* cells. Data show significant dysregulation of some of these genes in HPRT-deficient cells, most notably *Foxp1* whose the level of gene expression is significantly reduced in HPRT-deficient cells (Figure 2C). Conversely, it is significantly increased for *Pde10a*, *Arpp19* and *Rgs9* genes relative to control (Figure 2C). Previous studies have established that while Bcl11b drives the differentiation of MSNs during embryogenesis, its expression persists during adulthood, thus indicating that it may participate to the function and the maintenance of mature MSNs [20], [25]. Accordingly, we assessed the level of Bcl11b mRNA and protein expression in wild-type and HPRTKO mice striatum. Akin to the data derived from the striatal cells, our results show that both mRNA and protein level for Bcl11b are markedly reduced in the striatum of HPRTKO adult mice relative to their wild-type counterparts (Figure 2D & 2E).Furthermore, the data also show that the gene expression of some of other striatally-enriched genes like Foxp1, Ngef and PDE10A is significantly dysregulated in the striatum of HPRTKO mice (Figure 2F). In separate experiments, we have also confirmed blunted gene expression for Bcl11b in HPRT-deficient mouse neuronal cells differentiated from mouse embryonic stem cells (ESD3) and in human neuron-like cells derived from differentiation of human embryonic carcinoma cells (Kang and Guibinga, unpublished data).

### Altered DARPP-32(Ppp1rb) Expression and Signaling in HPRT-deficient Striatal (STHdh) Cells and in the Striatum of HPRTKO Mice

**Figure 3:**
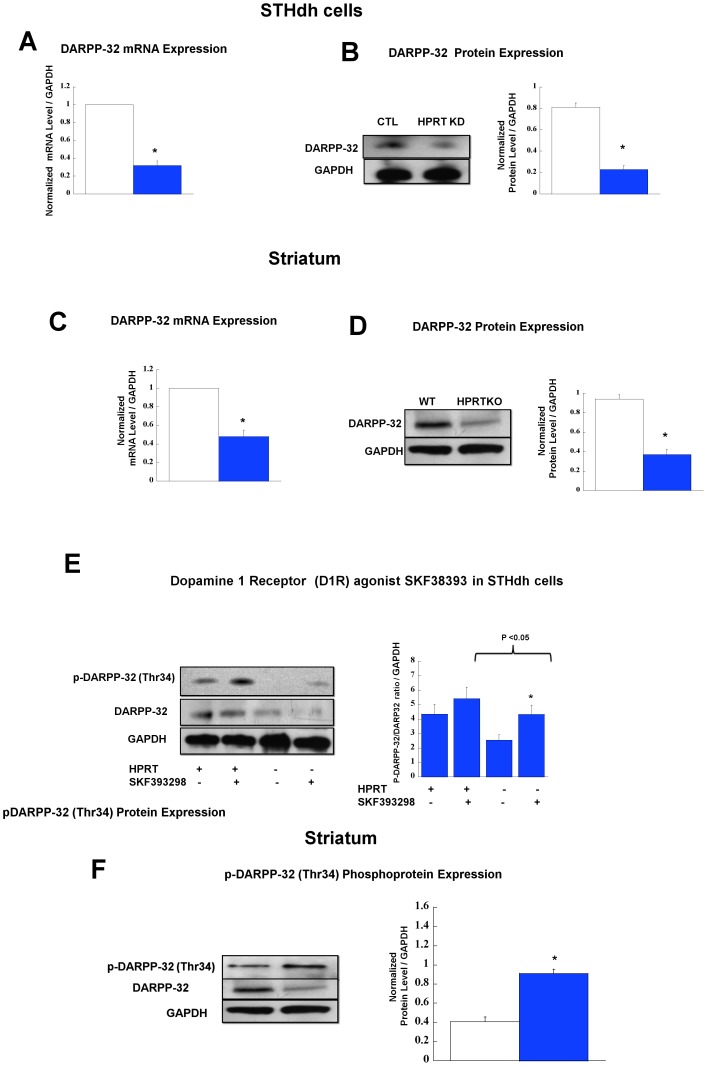
Gene and Protein Expression for *Ppp1r1b*/DARPP-32. (**A**) Gene expression for *Ppp1r1b*/DARPP-32 in control striatal STHdh cells (CTL, open bars) and HPRT-knockdown (HPRTKD, closed bars). Bars represent mean ±SEM (n = 4). mRNA expression is normalized to GAPDH mRNA level (*P<0.05, t-test). (**B**) Immuno-blot and quantification of DARPP-32 protein expression in striatal cells. The quantification bar graphs are shown as means ± SEM (n = 3, *P<0.05, t-test). (**C**) Gene expression for *Ppp1r1b*/DARPP-32 in the striatum of wild-type (WT, open bars) and HPRT knockout (HPRTKO, closed bars) mice. Bars represent mean ±SEM (n = 4). mRNA expression is normalized to GAPDH RNA level (*P<0.05, t-test). (**D**) Immuno-blot and quantification of DARPP-32 protein expression in the striatum of WT and HPRTKO mice. The quantification bar graphs are shown as means ± SEM (n = 3, *P<0.05, t-test). (**E**) DARPP-32 phosphoprotein (Thr34) expression in response to D1R (SKF393298) agonist treatment (50 µM). Immuno-blot and quantification of Thr34-DARPP-32 in control and HPRT-deficient striatal cells, the quantification bar graphs are shown as means ± SEM (n = 6, *P<0.05). (**F**) Thr34-DARPP-32 phosphoprotein expression in striatal tissue of WT (open bar) and HPRTKO (closed bar) mice (n = 3, *P<0.05).

We set out next to evaluate the expression of DARPP-32 in HPRT-deficient striatal cells and tissues, DARPP-32, a striatally-enriched signaling molecule that is encoded by the *Ppp1r1b* gene is known to be critical for striatal signaling and neurotransmission [26], [27]. Our focus on DARPP-32 stems from earlier research which demonstrates that Bcl11b knockout mice exhibit marked reduction of DARPP-32 in the striatum [24]. Our data show a significant down-regulation of *Ppp1r1b*/DARPP-32 at the mRNA and protein level in HPRT-deficient ST*Hdh* cells and striatal tissue derived from adult HPRTKO mice. (Figure 3A &3B) and (Figure 3C & 3D). Finally we also examined Thr34-DARPP-32 phosphorylation, the data show that p-DARPP-32 (Thr34) expression in response to D1R agonist (SKF39288) is significantly reduced in HPRT-deficient cells (Figure 3E). Conversely, the level of p-DARRP-32 (Thr34) expression is increased in HPRTKO striatal tissue relative to wild-type (Figure 3F).

### Gene Expression Analysis Bcl11b and Darpp-32 (Ppp1r1b) in LNS Patients

**Figure 4:**
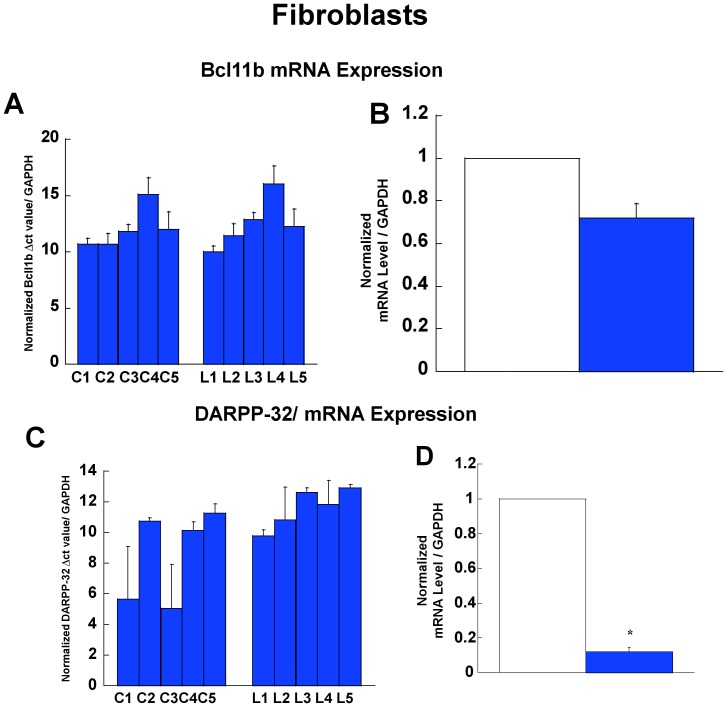
Bcl11b and *Ppp1r1b*/DARPP-32 in human fibroblasts. (**A**) *Bcl11b* expression in individual fibroblast samples from five control (normal) subjects (C1–C5) and from five patients with LNS (L1–L5). Δct values are normalized to control mRNA (GAPDH). (**B**) Normalized *Bcl11b* expression in controls (CTL) and LNS patients. (**C**) *Ppp1r1b/*DARPP-32 expression in individual fibroblast samples from five control (normal) subjects (C1–C5) and from five patients with LNS (L1–L5). Δct values are normalized to control mRNA (GAPDH). (**D**) Normalized *Ppp1r1b*/DARPP-32 expression in controls (CTL) and LNS patients (*P<0.05, *t*-test).

In order to demonstrate that the changes in *Bcl11b* and *Ppp1r1b*/DARPP-32 gene expression are not unique to murine cells or tissues, we comparatively examined the expression profile of Bcl11b and *Ppp1r1b*/DARPP-32 mRNA in primary culture of fibroblast cells derived from control (normal) and LNS patients. The data show that the level of Bcl11b gene expression is only slightly reduced in the fibroblast cells of LNS patients comparatively to control normal subjects (Figure 4A &4B). On the contrary, *Ppp1r1bB*/DARPP-32 shows marked down-regulation in fibroblasts derived from LNS patients comparatively to control normal subjects (Figure 4C & 4D).

### BDNF/TrkB Expression and Signaling in HPRT-deficient Striatal (STHdh) Cells and Tissues

**Figure 5:**
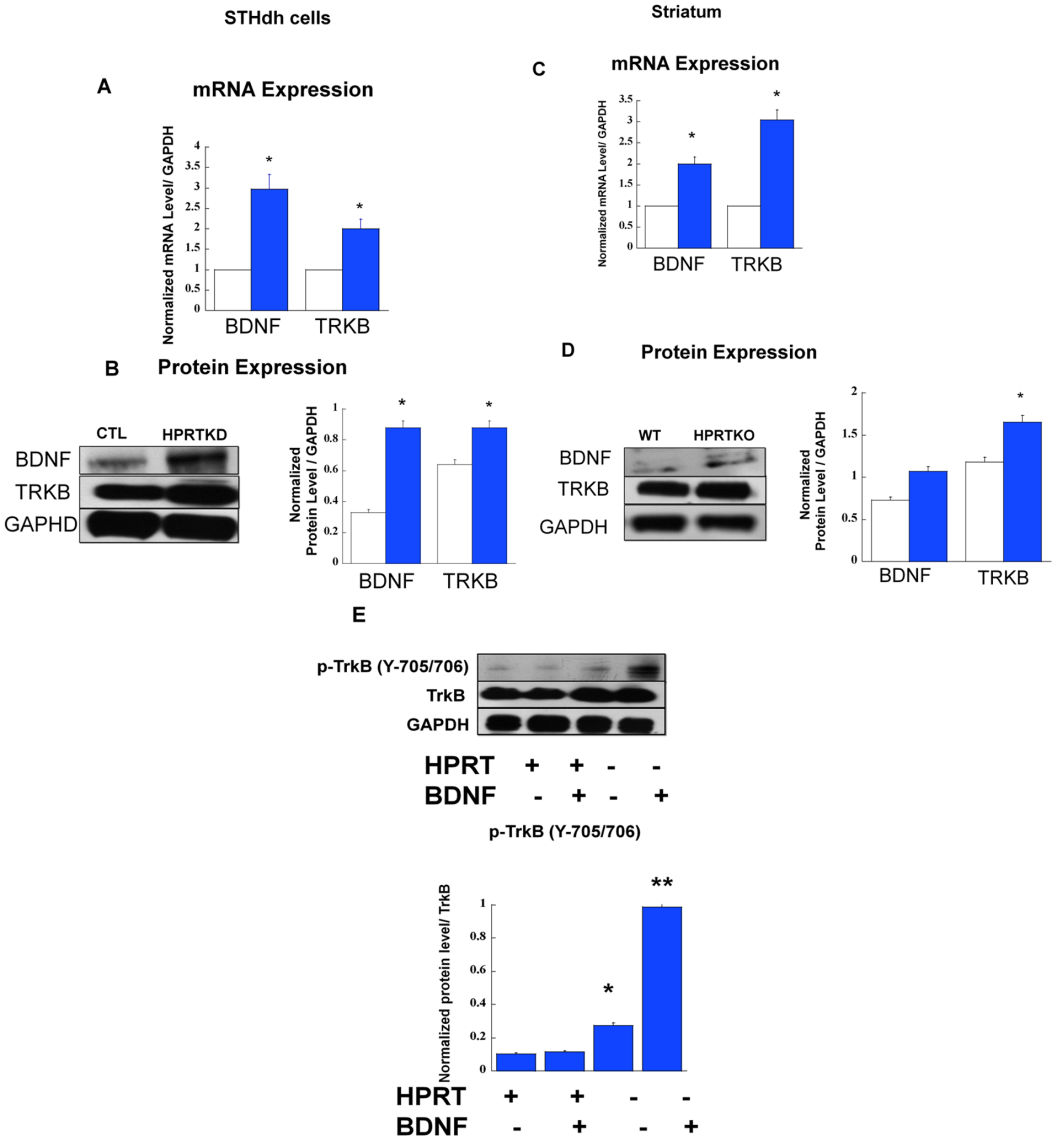
Gene and Protein Expression for BDNF and TRKB. (**A**) Gene expression for *Bdnf* and *Trkb* in control (CTL, open bars) and HPRT knockdown (HPRTKD, closed bars) striatal STHdh cells. Bars represent mean ±SEM (n = 4). mRNA expression is normalized to GAPDH RNA level (*P<0.05, t-test). (**B**) Immuno-blot and quantification of BDNF and TRKB protein expression in striatal cells. The quantification bar graphs are shown as means ± SEM (n = 3, *P<0.05, t-test). (**C**) Gene expression for *Bdnf* and *Trkb* in the striatum of wild-type (WT, open bars) and HPRT knockout (HPRTKO, closed bars) mice. Bars represent mean ±SEM of duplicate PCR measurements (n = 4). mRNA expression is normalized to GAPDH mRNA level (*P<0.05, t-test). (**D**) Immuno-blot and quantification of BDNF and TrkB protein expression in the striatum of WT and HPRTKO mice. The quantification bar graphs are shown as means ± SEM (n = 3, *P<0.05, t-test). (**E**) (Y705/706) TrkB phosphoprotein expression in response to BDNF treatment (10 ng/ml). Immuno-blot and quantification of Y705/706) TrkB phosphoprotein in control and HPRT-deficient striatal cells, the quantification bar graphs are shown as means ± SEM (n = 6, *P<0.05).

All the previous results clearly established that Bcl11b and DARPP-32 have a role in HPRT-deficiency. In the following experiments, we set out to assess BDNF expression and signaling in HPRT-deficient striatal cells and tissues, our reasoning was rooted in a recent report implicating Bcl11b in the regulation of BDNF signaling pathway [25], and also from the fact that BDNF is a known inducer of DARPP-32 expression in the striatum [28]. The data show increased mRNA and protein level for both BDNF and TrkB in HPRT-deficient striatal cells and in the striatum of HPRTKO mouse, comparatively to their respective control (Figures 5A, 5B, 5C and 5D). Subsequently, we sought to assess BDNF/TrkB signaling by treating control and HPRT-deficient striatal cells with BDNF (10 ng/ml). Immuno-blots show increased expression of phospho-TrkB- 705/706 (p-TrkB-705/706) upon BDNF treatment (Figure 5E). Using a phospho-tyrosine antibody which recognizes a broad range of phosphorylated tyrosine kinase motifs, we were also able to show that BDNF treatment also leads to increased expression of tyrosine-phosphorylated motifs (data not shown).

### HPRT-deficiency Confers Neuroprotective Effects against Reactive Oxygen Species (ROS)-mediated Neuronal Cell Death of Striatal Cells

**Figure 6:**
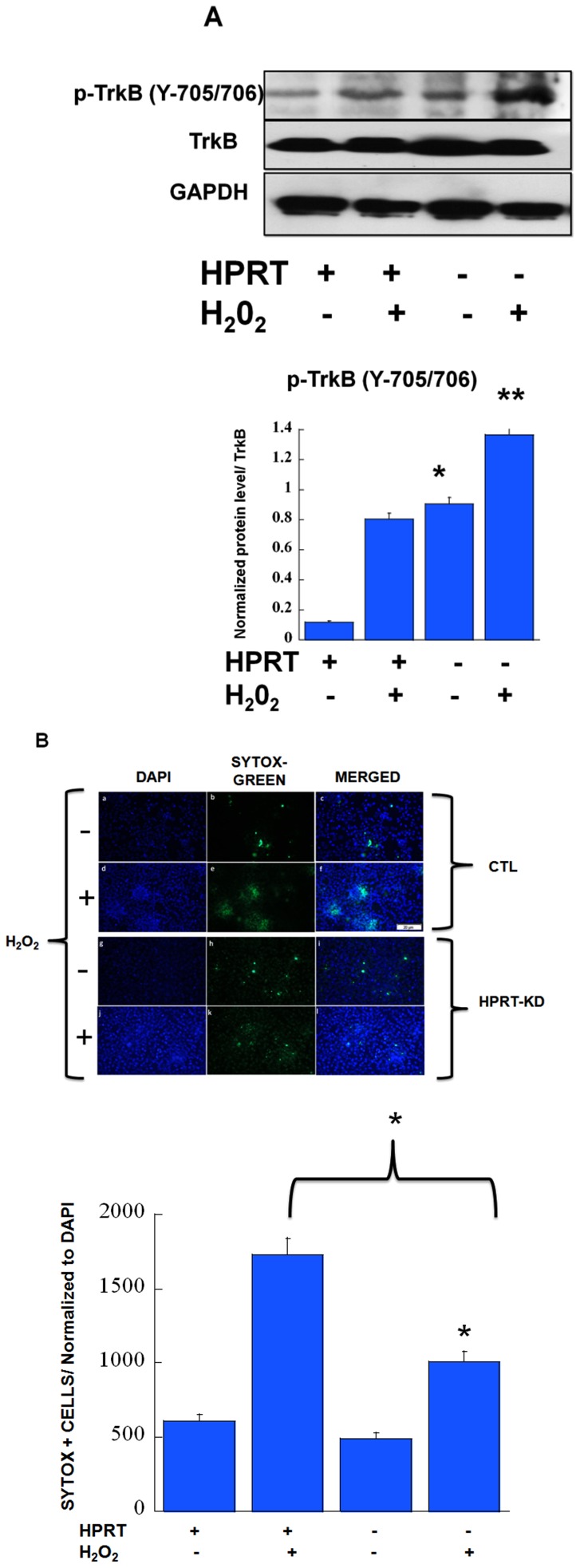
Effects of hydrogen peroxide-mediated cellular toxicity in HPRT-deficient striatal cells: (**A**) (Y705/706) TrkB phosphoprotein expression in response to hydrogen peroxide H_2_O_2_ treatment (100 µM). Immuno-blot and quantification of Y705/706 TrkB phosphoprotein in control and HPRT-deficient striatal STHdh cells. The quantification bar graphs are shown as means ± SEM (n = 6, *P<0.05). (**B**) Enhanced neuroprotective effects against reactive oxygen species (ROS)-mediated cell death in HPRT-deficient striatal cells. Control (CTL) and HPRT-knock-down cells (HPRTKD) were treated with hydrogen peroxide H_2_O_2_ for 4 hours and then exposed to Sytox green for 10 min. Figure shows microscopy images of DAPI staining and green fluorescence which is a measure of the overall cell death level. There is reduced green fluorescence in HPRT-deficient cells relative to control after stimulation with H_2_O_2_. (Bar scale, 100 µm). This is confirmed by the quantification of the number of Sytox green fluorescent cells as illustrated in the appended graph. Error bars represent mean ± SEM of duplicate measurements of two independent experiments (n = 4). The asterisks (*) represent statistical significance between H_2_O_2_ treated cells (p<0.05, t-test).

We next query as to whether the up-regulation of BDNF/TrkB signaling pathway in HPRT-deficiency confers neuroprotection to striatal cells against ROS-mediated neuronal cell death, given that BDNF/TrkB signaling is well known for its pro-survival properties [29]. Firstly, our data show that akin to BDNF treatment, exposure of HPRT-deficient striatal cells to hydrogen peroxide leads to enhanced expression of TrkB signaling (Figure 6A). Secondly, the HPRT-deficient cells have an increased resistance to H_2_O_2_/ROS-mediated cell death as evaluated by immuno-fluorescent imaging and appended quantification of cellular death by SYTOX-based assay (panel of images Figure 6B).

### Rescue of Bcl11b Expression in HPRT-deficient Striatal (STHdh) Cells

**Figure 7:**
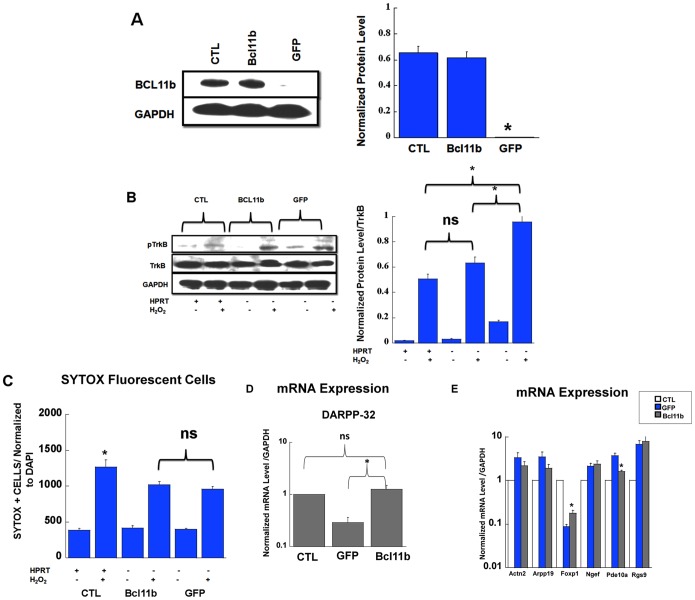
Restoration of Bcl11b expression in HPRT-deficient striatal cells: (**A**) Immuno-blot and quantification of Bcl11b expression in control (CTL) and HPRT-deficient striatal cells transfected with plasmid encoding Bcl11b (Bcl11b) and GFP (GFP). The quantification bar graphs are shown means± SEM (n = 3, *P<0.05, ANOVA, Tukey post-hoc test). (**B**) Immuno-blot and quantification of Y705/706 TrkB after H_2_O_2_ exposure for control (CTL), HPRT-deficient cells transfected with Bcl11b (Bcl11b) and GFP (GFP). The quantification bar graphs are shown as means ± SEM (n = 4, *P<0.05, ANOVA, Tukey post-hoc test). (**C**) quantification of the number of Sytox green fluorescent cells upon treatment of control (CTL) and HPRT-deficient cells transfected with Bcl11b (BCL11b) and GFP (GFP) plasmid. Data show that the rescue of Bcl11b expression in HPRT-deficient cells does not lead to added protection from cell death triggered by H_2_O_2_ (ns =  non significant, *p<0.05, ANOVA). (**D**) Gene expression of DARPP-32 in control (CTL), GFP and Bcl11b (rescued) transfected cells. Data show that the rescued expression of Bcl11b in HPRT-deficient cells leads to restoration of DARPP-32 expression in level similar to control, the data are shown as means ± SEM ((ns =  non significant, n = 4, *P<0.05, ANOVA, Tukey post-hoc test). (**E**) Gene expression for Actn2, Arpp19, Foxp1, Ngef, Pde10a, and Rgs9 in control (CTL, open bars), GFP (GFP, closed bars) and Bcl11b (Bcl11b, grey bars) transfected striatal cells. Bars represent mean ±SEM (n = 4). *P<0.05, ANOVA.

In order to determine the extent to which the down-regulated expression of Bcl11 in HPRT-deficient cells participates to aberrant TrkB signaling and the phenotype associated with superior protection against ROS-mediated neuronal cell death, we were prompted to rescue Bcl11b expression. HPRT-deficient cells were transfected with an expression plasmid encoding GFP (transfection control) or Bcl11b genes. Figure 7A shows robust Bcl11b protein expression in HPRT-deficient striatal cells transfected with the plasmid encoding Bcl11b versus the transfection control GFP. Additionally, the control cells along with the transfected ones were exposed to H_2_O_2_ in order to monitor TrkB signaling; the data show that rescue of Bcl11b leads to the diminution of p-TrkB overexpression (Figure 7B), however the lessening of pTrkB signaling was not sufficient to reverse or influence the phenotype of increased protection against H_2_O_2_-ROS-mediated cells death (see Figure 7C). Finally, as a last inquiry on the role of Bcl11b in driving the dysregulated expression of several of striatally-enriched genes including DARPP-32 in HPRT-deficient striatal cells, we examined their expression level in HPRT-deficient Bcl11b-rescued cells. Our data demonstrate that the rescued expression of Bcl11b in HPRT-deficient striatal cells corrects the dysregulation of *Ppp1r1b*/DARPP-32 gene (Figure 7D). In addition, for the other genes, the data show that the rescue of Bcll11b had only a significant effect on the gene expression *Foxp1* and *Pde10* (Figure 7E).

## Discussion

Biochemical and neuro-anatomical aberrations have long been described in the striatum of LNS and its murine model, the HPRTKO mice [30]. These alterations were believed to be in part responsible for HPRT-deficiency-related neurological deficits in LNS. Yet, there has been almost no studies examining the molecular and the cellular basis underlying these striatal abnormalities. In the present study, we have examined the expression and the signaling of key striatal genes and proteins, and have found that several of these genes and signaling pathways are aberrantly expressed, starting with the neurodevelopmental transcription factor Ascl1. We have previously demonstrated that Ascl1 expression is down-regulated in HPRT-deficient dopaminergic cell lines [10], hence implying that this transcription factor is important for dopaminergic neurodevelopment. However, the *Ascl1* gene is also known to be essential for the “pan-neurogenesis”, especially GABAergic neurons, including MSNs that make up the majority of neuronal cells in the striatum [11]. We have examined the gene expression of ASCL1 and some its transcriptional partners including FOXG1, DLX2 and GSX1 in HPRT-deficient striatal cells. We report that the *Ascl1* gene and protein expression are markedly blunted in HPRT-deficient striatal STHdh cells. In addition, the level of mRNA for FOXG1 and DLX2, two transcription factors important for proper striatal GABAergic specification is also significantly reduced in the same HPRT-deficient striatal cells. Of interest, is that mutations in the FOXG1 gene are associated with congenital variants for Rett Syndrome (FOXG1 syndrome), a severe neurodevelopmental disorder that is characterized by mental retardation and jerky movement [31]–[33]. While it remains to be determined whether or not the reduced level of FOXG1 gene expression in HPRT-deficiency is partly responsible of neuropathogenesis of LNS, our data suggest that there may be neuropathogenic commonalities between LNS and FOXG1 syndrome.

In the striatum, HPRT-deficiency leads to the dysregulation of several transcription factors; however we elected to examine the level of expression of Bcl11b. Our choice was justified by the fact that this transcription factor, unlike Ascl1 has a well documented role in controlling striatal gene expression and transcriptional dysregulation in a disease model [20], [25]. We showed Bcl11b mRNA and protein expression are significantly decreased in both striatal cells and the striatum of HPRTKO mice. Since Bcl11b is known to drive the expression of hundred of striatally-enriched genes [20], [25], we set out to analyze the level of expression of six of those genes, some of them are differentially expressed between control and HPRT-deficient striatal cells and tissue, among which Arpp19, a cAMP regulated phosphoprotein involved in the control of mitosis [34], and whose the expression is reduced in the brains of Alzheimer and Down Syndrome patients [35]. Also the transcription factor Foxp1, which is known to have a prominent role in the CNS development and whose the expression is dysregulated in Huntington disease and Autism [36]–[39]. In addition, our data shows increased mRNA level for Rgs9, which is highly enriched in the dopamine receptor regions of the striatum, where it modulates signaling pathways associated to GPCR [40]–[42]. Finally, we also showed that PDE10A mRNA is increased in HPRT-deficient striatal cells. PDE10a is highly enriched in the striatum and is known to be important in regulating striatal cell signaling [43]–[45]. The demonstration in this study of increased gene expression of PDE10a in HPRT-deficient striatal cells is consistent with our recent demonstration of enhanced PDE10a expression and activity in HPRT-deficient neuronal cell lines [21]. Collectively, these data suggest that cAMP related signaling via Arpp19, Rgs9, or PDE10 are severely affected in HPRT-deficiency, thus confirming our two recent reports on cAMP–related signaling dysregulation in various HPRT-deficient human and mouse cells and tissues [21], [46].

This study shows that *Ppp1r1b*/DARPP-32 expression is reduced in HPRT-deficient striatal cells and in the striatum of adult HPRTKO mice (Figure 3A–3D). Furthermore, we demonstrate that the expression of phosphorylated DARPP-32 (at position Thr34) is blunted in HPRT-deficient striatal cell lines relative to control in response dopamine receptor 1 agonist SKF38393 (Figure 3E); Paradoxically phosphorylated DARPP-32 (Thr34) is increased in the striatum of adult HPRTKO (Figure 3F). We elected to study DARPP-32 expression and signaling, since previous studies indicate DARPP-32 is severely blunted in Bcl11b knockout mice [24]. DARPP-32 is a striatally-enriched phosphoprotein and is central to the neurotransmission of the striatum [47]. Among the many functions of DARPP-32, is its role as a transducer of cAMP/PKA signaling. The phosphorylation of DARPP-32 by PKA on Thr34 converts this protein into an inhibitor of protein phosphatase PP-1, which under normal conditions de-phosphorylates several cellular targets, including glutamate and GABA receptors, as well as many ion channels, transcription factors and phosphatases and kinases [27], [47], [48]. Our findings as well as what we know to be the broad role of DARPP-32 in striatal neurotransmission lead us to believe that DARPP-32 plays a critical role in the etiology of LNS neuropathogenesis.

Our demonstration of blunted expression of agonist-induced/PKA-mediated DARPP-32 (Thr34) is consistent with our recent report of reduced PKA signaling in HPRT-deficient neuronal cell lines upon agonist-induced cAMP production [21]. Paradoxically, the increase of p-DARPP-32 (Thr34) in HPRTKO striatum is also consistent with the phenotype of HPRTKO mouse model, which displays an heighten sensitivity to amphetamine-induced locomotor activity [49]. The heighten sensitivity to amphetamine-induced locomotor activity is an indicator of increase D1-like receptor activity [50], [51], whose the signaling is transduced through DARPP-32 [52]. Taken together, the differences in DARPP-32 (Thr34) expression between HPRT-deficient striatal neural stem cells and adult striatal tissues likely reflect the distinct cellular and signaling make up of this immortalized striatal cell and mature striatal neurons.

DARPP-32 was initially identified as the main target of dopamine-activated adenylyl cyclase in the striatum [26], [53], this important phosphoprotein is well known to be a central integrator of neuronal signaling that converges onto dopamine-receptive neurons in the striatum via the action of neurotransmitters, neuromodulators and neuropeptides [47]. DARPP-32 neuronal signaling is critical for motivated behavior, learning and memory and is also associated to pathogenic conditions such as schizophrenia and addictions, DARPP-32 has been considered for therapeutic targeting in various neurobehavioral disorders [54]. Pending a more complete delineation of the role of DARPP-32 in the etiology of the LNS, the findings of our study raise the possibility that targeting DARPP-32 in this disease may also be a viable therapeutic strategy.

We also set out to test the level of gene expression of Bcl11b and DARPP-32 in fibroblast cells derived from LNS patients (Figure 4). These experiments were meant to infer that the dysregulated expression of these two key genes also extend to LNS subjects, and could possibly be used as biomarkers of HPRT-deficiency neurological phenotype in humans. While we are mindful that fibroblasts and neurons have a different genomic and cell signaling make up; it is worth mentioning that HPRT-deficient fibroblasts derived from LNS patients have often confirmed gene expression alterations relevant to neuronal pathways [15], [46].

Bcl11b and its target genes have role in BDNF signaling, which is itself an inducer of DARPP-32 [25], [28], this led us to examine the involvement of BDNF and its receptor tropomyosin related kinase B (TrkB) in the striatal gene expression. Our data show that BDNF and TrkB expression are increased in HPRT-deficient striatal cells and in the striatum of HPRTKO mice (Figure 5); we also show enhanced level of TrkB phosphorylation (Y705/706) in response to BDNF and Hydrogen peroxide (Figure 5 & 6). Our study reveals that DARPP-32 expression is down-regulated despite up-regulation of BDNF; this discrepancy may be attributed to the severe down-regulation of Bcl11b. In fact restoration of Bcl11b expression in HPRT-deficient striatal cells also re-establishes DARPP-32 gene expression (Figure 7D). This suggests that Bcl11b may directly or indirectly control DARPP-32 expression. These data are consistent with previous research which shows that Bcl11b knockout mice have marked down-regulation of striatal DARPP-32 [24]. Over all, we noted that control STHdh cells were almost non responsive to BDNF treatment, we can only speculate that this difference may be attributed non to low level of BDNF receptor expression in control cells comparatively to the HPRT-deficient ones.

BDNF/TrkB signaling plays a critical role during early brain development and is associated with the regulation of synaptogenesis, synaptic plasticity and neurogenesis in mature brain [55]. From a broader neuropathogenic standpoint, BDNF-related signaling is blunted in several neurological disorders, specifically prominent diseases like Alzheimer, Parkinson and depression [56]–[58]. Conversely, BDNF is increased in autism spectrum disorders (ASD) [59]. While the relevance of our findings to the neuropathogenicity of LNS remains to be fully established, our results suggest that BDNF dysregulation may be part of LNS-neural related abnormalities. Thus in the context of LNS, BDNF may be amenable to therapeutic modulation as suggested for other neurological disorders [60].

One of the well recognized actions of BDNF in the CNS is its neuroprotective role against the various neuronal injuries caused in neurodegenerative diseases or stroke. In present study we wanted to determine whether the enhanced expression of BDNF/TrkB signaling in HPRT-deficient striatal cells confers added protection against hydrogen peroxide-mediated cell death through oxidative stress. Our data reveal that HPRT-deficient striatal cells are less susceptible to neuronal cell death induced by hydrogen peroxide comparatively to the control, HPRT-positive cells (Figure 6). While HPRT-deficiency affects striatal development programming, it does not completely blunt the generation of neurons, nor does it affects the survival of striatal neurons. The striatum in LNS and HPRT-knockout mice contain normal number of striatal cells, and unlike Huntington disease, show no evidence of striatal cellular degeneration [16]. We do not know yet the broad significance of the up-regulation of pro-survival pathways in HPRT-deficiency; but we suspect that the molecular and the cellular adaptations caused by the purine metabolic defects in LNS/HPRT-deficiency generate purine metabolites such as uric acid that could bring about the augmentation of pro-survival signaling pathways and lead to the observed survival advantage. In fact, uric acid is known for its antioxidant and neuroprotective properties [61]. Recent studies have identified uric acid as protector of 1-methyl-4-phenyl-pyridinium (MPP(+))-induced degeneration of dopaminergic neurons in vitro [62]. In addition, clinical and epidemiological data have shown that uric acid is a predictor for reduced risk and favorable progression in Parkinson Disease (PD) [63].

The rescue of Bcl11b expression in HPRT-deficient striatal cells, while it partly restores BDNF/TrkB signaling to the level of control (Figure 7B) had no significant effect in altering cellular neuroprotection of HPRT-deficient cells against peroxide-hydrogen-mediated cell death (Fig. 7C). This data suggest that BDNF/trkB may not the sole factor responsible for the neuroprotective phenotype displayed by the HPRT-deficient striatal cells; and as mentioned above the purine metabolic defect (uric acid) may be in part responsible of this neuroprotective phenotype.

**Figure 8:**
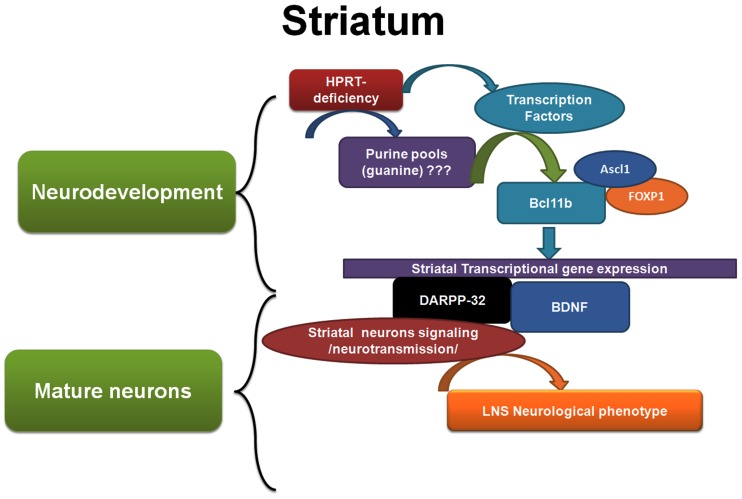
Schematic and summary model of HPRT-deficiency role in dysregulating striatal gene expression and signaling. HPRT-deficiency alters the purine pool; and via mechanisms that are still ill-defined, these alterations affect the gene expression of keys striatal-enriched transcription factors such as Ascl1, Bcl11b and Foxp1, notably during neurodevelopment. Later in mature neurons, these transcription factors control (directly or indirectly) the expression of several genes among them, *Darpp-32* and *Bdnf*, whose the dysregulation affects striatal neurons signaling and neurotransmission, thus contributing to LNS neurological phenotype.

Collectively, our study shows that HPRT-deficiency caused dysregulation of genes responsible for striatal neurodevelopment particularly during embryogenesis affecting the expression of transcription factors such as Ascl1, Bcl11b and Foxp1. These transcription factors through their pleiotropic effects regulate the expression of hundred of genes essential for the function of striatal progenitor cells and mature striatal neurons. Among the dysregulated genes are those vital for neurotransmission such as ppp1r1b/DARPP-32 (see figure 8). While the initial event(s) (purine-related or otherwise) that leads to the cascade of molecular and cellular aberrations in HPRT-deficiency remains to be identified. This study has unraveled keys genes and signaling pathways that may have an immense importance in the understanding of the etiology of LNS. Some of these genes and pathways (DARPP-32 and BDNF) have already been well characterized in other neurological diseases. Therefore, in the context of HPRT-deficiency our findings open the doors to additional investigations that take into account the wealth of information accumulated on these dysregulated pathways in other disease models and how they contribute to disordered brain function of LNS patients. Ultimately we hope that the development of therapeutic strategies will lead to disease-modifying therapies for LNS and other neurological diseases.

## Supporting Information

Table S1 List of Primers.
(PDF)
