# Supplementary material for: Striatal Neurodevelopment Is Dysregulated in Purine Metabolism Deficiency and Impacts DARPP-32, BDNF/TrkB Expression and Signaling: New Insights on the Molecular and Cellular Basis of Lesch-Nyhan Syndrome
Source: PLoS One. 2014 May 7;9(5):e96575. doi: 10.1371/journal.pone.0096575 (PMC4013014; doi:10.1371/journal.pone.0096575)
Supplement: Table S1 — List of Primers. (PDF) [file pone.0096575.s001.pdf]

Table 1s: List of Primers

PRIMERS	SEQUENCES	AMPLICON SIZE
m-HPRT-F	TGTTGTTGGATATGCCCTTGAC	
m-HPRT-R	GGCCACAGGACTAGAACACC	180
m-ASCL1-F	TGACTTTTGCGGCTGCTTTC	
m-ASCL1-R	GCAGAAGGAGGCAGAGTTGT	200
m-FOXG1-F	ACTGTTTGGGTCTGTGCTCG	
m-FOXG1-R	GTAGCAAAAGAGCTTCCTGCG	163
m-DLX2-F	ACCCAGCGGTATTGGAAGTG	
m-DLX2-R	CCAGGCTTGGCACTAAAGGA	117
m-GSX1-F	GACATGTCGCGCTCCTTCTA	
m-GSX1-R	ATGAAGAAATCCGGCCCTGG	101
m-actinin2-F	CCCAGCGCCATGAATCAGATA	
m-actinin2-R	ATGTTCTCGATCTGGGTGCC	194
m-RGS9-1-F	CGTGTATACAGGGACCTGCG	
m-RGS9-1-R	AGAACCCTCCAAAGCCACTC	157
m-ARPP19-F	GTCTTGCCTCCCGGTAATCT	
m-ARPP19-R	ATCAGACCCTCCAGGCTTTTG	197
m-NGEF-F	TGCTTATGTGGGGGACACAC	
m-NGEF-R	GCCAGAAGTTCCTGGGTGTT	113
m-BCL11b-F	CACCCACGAAAGGCATCTGT	
m-BCL11b-R	GCACGCAGAGGTGAAGTAATC	136
m-FOXP1-F	TTTCCGAGGGAACCCTTTCG	
m-FOXP1-R	GGAGCCCTTTAGGCTAGCAG	105
m-PDE10A-F	ATACCACCCGGGATGAAGGA	
m-PDE10A-F	AAGAACAGACTGGATGCGGG	186
m-PPP1R1B-F	CGACAGGTGGAGATGATCCG	
m-PPP1R1B-R	CACAGGGGTTGGGTCTCTTC	156
h-PPP1R1B-F1	ACTTCTCACAAGGACTGGGTG	
h-PPP1R1B-R1	TCTGGTGAGGAGTGCTCTGA	182
m-BDNF-F	GGGAAATCTCCTGAGCCGAG	
m-BDNF-R	AGCTTTCTCAACGCCTGTCA	171
h-GAPDH-F	GACAGTCAGCCGCATCTTCT	
h-GAPDH-R	GCGCCCAATACGACCAAATC	104
m-GAPDH-F	CTCCCACTCTTCCACCTTCG	
m-GAPDH-R	TAGGGCCTCTCTTGCTCAGT	193
m-TRKB-F1	CCAAGTTTGGCATGAAAGGC	
m-TRKB-R1	TGCCAAAGTACTGGGGGTTT	101
